# Patients’ and Caregivers’ Experiences Navigating the Burden of Atopic Dermatitis in Argentina

**DOI:** 10.3390/medicina60040584

**Published:** 2024-03-31

**Authors:** Korey Capozza, Michelle Tu, Alan Schwartz, Jodi L. Johnson, Mónica Ladner

**Affiliations:** 1 Global Parents for Eczema Research (GPER), Santa Barbara, CA 93101, USA; michelle@gper.org; 2 Departments of Medical Education and Pediatrics, University of Illinois, Chicago, IL 60607, USA; 3 Departments of Pathology and Dermatology, Northwestern University, Chicago, IL 60611, USA; jodi-johnson@northwestern.edu; 4 Asociación de Dermatitis Atópica Argentina (ADAR), Buenos Aires C1424BDV, Argentina; pacientes.adar@gmail.com

**Keywords:** atopic dermatitis, eczema, caregiver burden, disease burden, financial stress, health-related quality of life, shared decision making, patient education

## Abstract

*Background and Objectives***:** Little is known about patients’ and caregivers’ experiences with atopic dermatitis (AD) in Argentina, so a survey was administered to learn more. *Materials and Methods*: A 53-item anonymous survey was administered in Spanish to adult AD patients (*n* = 334) and caregivers (*n* = 339) of pediatric AD patients in Argentina (total *n* = 673). Demographics, healthcare provider information, financial burden, disease severity, disease burden, level of disease-specific education, and experience with shared physician/patient decision making were collected. Linear and logistic regression models were used for statistical comparisons. *Results*: Survey respondents were overwhelmingly female (90.8%), as was the overall patient population (72.8%). Patients were seen mostly by healthcare specialists (66.8% dermatologists, 13.5% pediatricians, 7.7% allergists, and 7.2% general practitioners). Only 2.8% of respondents reported no symptoms, while 33.3%, 52.4%, and 11.5% reported mild, moderate, and severe AD disease, respectively. Anxiety/depression and pain/discomfort were the most impactful on respondents’ quality of life. Caregivers of children with moderate to severe AD and adult patients with severe AD reported a significant financial burden, including using savings or not purchasing food or other essentials to afford medical care. Few people reported receiving disease-specific education or having their own treatment priorities taken into consideration. For adult patients, receiving disease education and being asked about treatment priorities were associated with higher treatment satisfaction and AD control. *Discussion*: Mental health, pain/discomfort, and financial worries are the most important burdens for adult AD patients and caregivers of children with AD in Argentina. We recommend prioritizing disease-specific education and shared decision making to improve AD care in Argentina.

## 1. Introduction

The prevalence of atopic dermatitis (AD) in pediatric patients in Argentina has been observed at 9.7% and 7.8% among young children 6 months to 6 years of age [1] and 5% among children with a median age of 10 [2]. The prevalence of adult AD in Argentina is just below 5% [3]. Among adults in Argentina, the highest AD prevalence was reported in patients 35–44 years of age, and significantly more females than males were diagnosed with AD [3]. Only a few studies have examined the burden of AD among citizens of Argentina with respect to age, sex, type of physician managing their disease, quality of life, symptom burden using available treatment approaches, treatment satisfaction, and financial burden [3,4,5]. As patient disease-specific education and shared decision making between physicians and patients may improve treatment adherence, treatment satisfaction, and outcomes, the extent of disease-specific education and shared decision making in Argentina also needs to be studied [6].

Healthcare disparities exist throughout Latin America with regard to access to medical care, access to medical specialists, access to specific medications, treatment understanding (disease-specific education/shared decision making), and treatment outcomes based on rural versus urban dwelling, socioeconomic status, and other factors [5]. Some of the front-line AD medications used in other countries are not approved for use in Argentina, such as methotrexate, azathioprine, and mycophenolate (all can be used for AD off-label). Even if drugs are approved for use in AD treatment in Argentina, as in the case of cyclosporin, dupilumab, and upadacitinib, patients may have limited access through public clinics. Medications may also not be financially covered through the public healthcare system [5]. Phototherapy is not available in many clinics and can be very costly to obtain [5]. In Argentina, the percent of AD patients receiving treatment was reported to be the lowest (64.6%) compared to 15 other countries surveyed, including the other Latin American countries Brazil (80.3%), Mexico (73%), and Columbia (73.5) [3]. According to PO-SCORAD scores in Argentina, AD patients receiving treatment had moderate to severe AD [3].

Previous studies have reported that general practitioners are trained to recognize the most prevalent diseases found in their geographical area. Because of this, AD is not typically diagnosed by general practitioners [5]. To effectively find treatments that lead to symptom control for AD, patients need to access specialists [5]. Out of 495 Argentinian AD patients surveyed, over 74% reported having their AD diagnosed by a dermatologist [3]. Allergy and Dermatology societies from Latin America reported 8 dermatologists and 5.7 allergists to 100,000 inhabitants in Argentina [5,7]. While there has not been a report about the distribution of dermatologists across the country, the time to diagnosis of AD was lower in cities compared to outlying areas of the country by up to six years [4,7], perhaps indicating that people in cities had better access to specialists. Even with specialists, the struggle to find appropriate therapies to control treatments can be ongoing. Previous surveys have reported that 40% of patients and their caregivers in Argentina are dissatisfied with their treatments [4,5].

A recent report described the impacts of AD on quality of life in Argentina using a web-based survey of 1650 AD pediatric and adult patients [4]. Respondents were largely from Buenos Aires City (17%) and the surrounding province (40%), 6% of respondents were from Cordoba, and 6% from Santa Fe, while 31% of respondents came from the rest of the country. The surveyed AD patients stated that mood alterations including frustration and anger, increased stress, reduced sleep, needing to change their daily routine and cancel plans, pain, and economic/financial impact were all factors that reduced their quality of life [4]. Sixty percent of patients reported being treated with topical corticosteroids, and the only other treatments reported were emollients or special soaps rather than any of the more advanced topical or systemic treatments that have emerged in North America, Europe, and Asia over the past decade [4]. One quarter of the patients included in this survey did not have health insurance and 34% of the total surveyed patients reported spending 20% of a minimum wage salary out-of-pocket on their AD-related health costs [4].

Since few previous surveys about the AD experience in Argentina exist, we aimed to add to and confirm previous findings, to reduce knowledge gaps, and to highlight challenges faced by AD patients and their caregivers in Argentina using a survey of adult AD patients and caregivers of pediatric AD patients.

## 2. Materials and Methods

### 2.1. Ethical Considerations, Prior Survey Use, and Survey Promotion in Argentina

This research was reviewed and approved by Advarra Institutional Review Board (Pro00055632) on 6 July 2022. Patients provided informed consent for participation in the survey and all procedures were conducted according to ethical guidelines. A 53-item anonymous survey designed for adult patients with AD and caregivers of children with AD has been reported previously after being administered across eight countries [8]. Because each question was optional, some respondents chose not to answer some questions. We included these partial respondents in analysis whenever they answered the relevant question to avoid biases associated with listwise deletion. Throughout this report, we included the number of respondents that did answer the question and were, therefore, included in the analysis for that question in the text and figure legends.

The survey included questions related to demographic characteristics, satisfaction with treatments, type of provider for AD care, experience with patient centered medical care practices, long-term symptom control, the burden of disease, health-related quality of life, and financial impacts. The domains and measurement tools for the survey were prioritized and selected by a committee of representatives from 11 patient organizations in the original eight countries (Australia, Canada, Denmark, Germany, France, Italy, United Kingdom, and United States). The survey had originally been conducted in five languages, including Spanish, and was administered in Argentina in Spanish. The survey was promoted through social media and communication channels of the Asociación Civil de Dermatitis Atópica Argentina, Argentina’s largest patient advocate organization. A stipend totaling US $100 was given to the Asociación Civil de Dermatitis Atópica Argentina, and survey participants could choose to enter a drawing for the US $100 to be delivered in the form of a local gift card.

### 2.2. Survey Characteristics

Demographic questions were used to identify the respondent’s age and sex. Caregivers or adult patients reported the patient age and sex, as well as self- or caregiver-reported information on the patient’s AD severity (“clear [sin dermatitis atópica or without symptoms]”, “mild [leve]”, “moderate [moderado]”, or “severe [grave]”), type of health care provider responsible for AD care (i.e., dermatologist, allergist, pediatrician, or general practitioner), and how they primarily paid for AD-related medical care (public health care system, private insurance, or personal finances).

We used existing validated measures for AD whenever possible. For example, long-term control of AD symptoms was measured using the AD Control Tool (0–100, with >70.8 indicating “adequate” control) [9]. General health-related quality of life was measured using the EQ-5D questionnaire, which is a widely used approach to measure health-related information and has over 150 translations available [10]. The EQ-5D questionnaire’s descriptive system captures the respondent’s level of impairment in 5 dimensions (mobility, selfcare, usual activities, pain/discomfort, and anxiety/depression). The ratings for this measure range from 0 (equivalent to death) to 1 (perfect health) and can be interpreted as the proportion of one’s remaining expected years of life that the individual would be willing to trade in exchange for perfect health. For example, a health state with a utility of 0.7 implies a willingness to trade 30% of one’s remaining life to be restored to perfect health.

When validated instruments were not available, we searched for survey questions that have been validated for other diseases and were available from publicly available data sources. For example, we measured satisfaction with treatments with an adapted version of the Psoriasis Satisfaction Survey, an 8-item questionnaire rated on a 5-point “agreement” scale (1: “I do not agree at all”; 5: “I agree completely”), with higher values indicating greater satisfaction. For the remaining topics of interest, we developed new measures. For example, to assess financial impact, we asked respondents whether and how the financial demands of treating AD affected their spending and saving decisions; respondents could select 1 or more option: “I had to use savings”; “I had to borrow money”; “I had to spend less on food and other essential expenses”; “I had to spend less on nonessential expenses”; or “Eczema (including treatments) did not affect my finances”.

Finally, we considered two dimensions of patient-centered care: training on AD self-management and shared decision making. We asked about disease-specific training with a series of three yes/no questions: “Has any health care provider ever offered you training on how to manage eczema or its symptoms?”; “Has any health care provider ever suggested you attend an eczema training program for patients and caregivers that happens after the office visit?”; and “Did the program that was recommended consist of multiple sessions totaling 6 h or more in length?” We assessed shared decision making with 2 yes/no questions: “At your most recent visit to the doctor, did the health care professional whom you see for AD ask about your priorities for your AD care?” and “Did the health care provider who you see for eczema include any of your priorities when making treatment recommendations?”

### 2.3. Statistics

Comparisons were made using linear and logistic regression models. Statistical analyses were conducted using R 3.6 (R Core Team, Vienna, Austria) and *p* ≤ 0.05 was considered statistically significant.

## 3. Results

### 3.1. Demographic Characteristics

Survey respondents included 339 caregivers answering the survey on behalf of their child and 334 adult AD patients answering the survey for themselves (*n* = 673 total). The mean age of survey respondents was 41 years with a range of 18–80 years old. Respondents were overwhelmingly female (90.8% female, 9.1% male, and 0.1% other). The mean age for patients was 27 years old with a range of 1–22 years for pediatric patients and 18–80 for adults. Overall, patients were 72.8% female, 27.1% male, and 0.1% other, consistent with previous reports that the majority of diagnosed AD patients in Argentina are female [3]. The sex of the pediatric population of patients was not statistically different with 45.4% being male and 54.3% being female compared to adult patients who were 91.3% female and 8.7% male (Table 1).

### 3.2. Type of Healthcare Provider

As was reported in previous studies [3,5], the majority of AD patients were being seen by a dermatologist (444 or 66.8%), while 90 (13.5%) were seen by a pediatrician, 51 (7.7%) were seen by an allergist, and 48 (7.2%) were seen by a general practitioner. Only 32 (4.8%) reported being seen by another type of practitioner. More adult patients reported seeing a primary care provider or general practitioner compared to pediatric patients (13.7% vs. 0.9%, respectively) (Table 1).

### 3.3. Disease Severity and Health-Related Quality of Life

**Figure 1:**
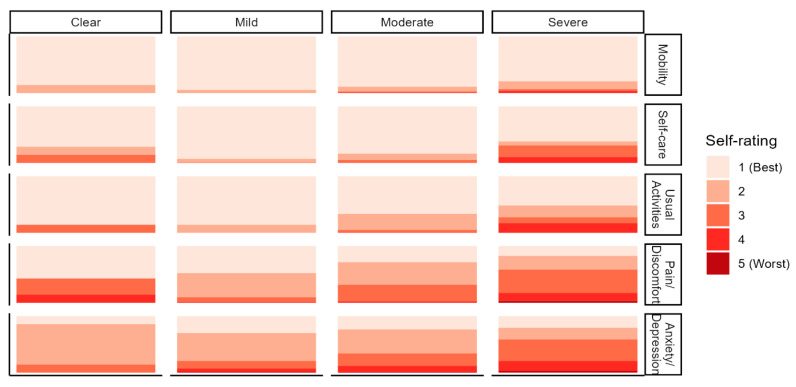
EQ-5D results indicating quality of life impacts of AD on adult patients in Argentina. Only the adult patients (*n* = 332) completed the EQ-5D. Clear means without symptoms. Pain/discomfort and anxiety/depression were worse than mobility, self-care, and ability to complete usual activities irrespective of severity.

The severity of diseases was self-reported. Overall, only 19 respondents (2.8%) reported the patient had clear disease (without symptoms), 223 (33.3%) reported mild disease, 351 (52.4%) reported moderate disease, and 77 (11.5%) reported severe disease (Table 1). The overall results of the EQ-5D questionnaire for Argentina showed the average score to be 0.83 (95% CI, 0.82–0.85) meaning adult patient respondents were willing to trade 17% of their remaining lifespan in order to live in perfect health. These values are similar to those reported in Germany and Italy in the eight-country comparison study, which were the countries with the highest quality of life using this scale [8]. Australia, Canada, Denmark, France, United Kingdom, and United States reported being willing to trade more lifespan for health, meaning their perception of quality of life was worse [8]. For comparison, the average EQ-5D score of these eight countries was 0.72 (95% CI, 0.65–0.78), which implies that patients with AD would trade 28% of their remaining life expectancy to be restored to perfect health. Pain and discomfort, as well as anxiety/depression, were of higher burden than mobility, self-care, and the ability to perform usual activities in Argentina (Figure 1).

### 3.4. Long-Term Control of Symptoms

**Figure 2:**
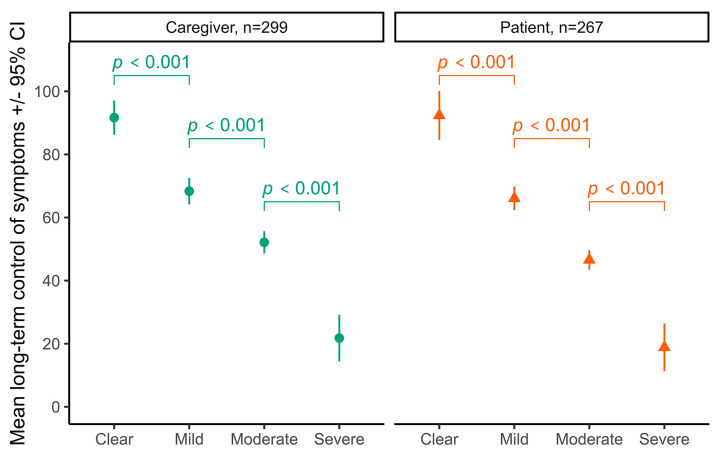
Long-term control of AD symptoms in patients. Long-term disease control significantly decreased by severity both when caregivers self-reported severity on behalf of their children and in adult patients (severity main effect F(3,558) = 59.4, *p* < 0.001). Clear means without symptoms. Results were compared using two-way ANOVA.

The long-term control of AD symptoms was measured using the AD Control Tool (0–100, with >70.8 indicating “adequate” control) [9]. Long-term control decreased as disease severity increased both in the caregiver group and in the adult patient group. Only the responders reporting clear (without symptoms) to mild reported “adequate” control (Figure 2).

### 3.5. Financial Burden

**Figure 3:**
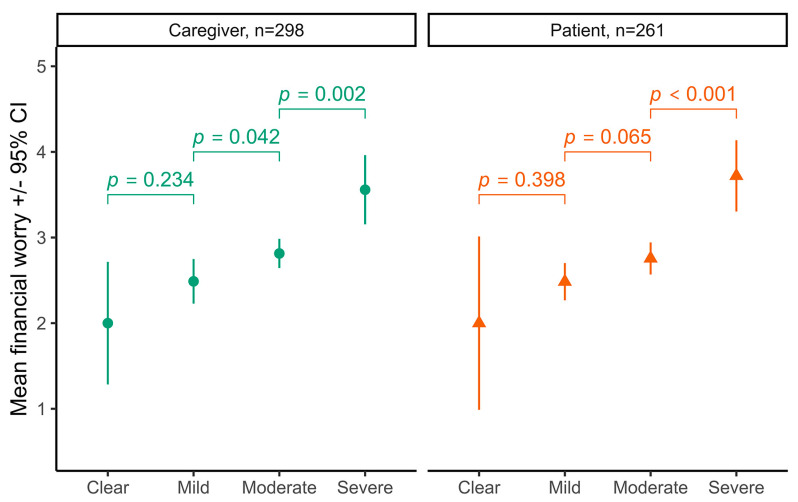
Financial worry of respondents. Financial worry increased by disease severity in both the caregiver group and the adult patient group (severity main effect F(3,551) = 10.1, *p* < 0.001). Clear means no symptoms.

The majority of participants in the survey identified financial worries as being a consistent part of their experiences with AD. A total of 559 out of the 673 survey participants responded to the question about financial worry, with 14% responding they were not at all worried, 10% responding they were extremely worried, and 75% indicating they were mildly to moderately worried. Only 109 survey participants answered the question on the impacts of AD care costs on their budgets. Those that did respond most commonly reported using less money for non-essentials (57%), using savings (44%), or spending less on food or other essentials (33%). The financial worry of survey respondents increased according to the patients’ severity of disease (Figure 3). Caregivers of pediatric children with moderate to severe AD had significantly higher financial worry than caregivers of children with no symptoms or mild disease. Adult patients with severe AD reported significantly higher financial worry than patients with clear (no symptoms), mild, or moderate AD.

### 3.6. Patient-Centered Medical Care, Shared Decision Making, Disease-Specific Education, and Association with Disease Control and Treatment Satisfaction

**Figure 4:**
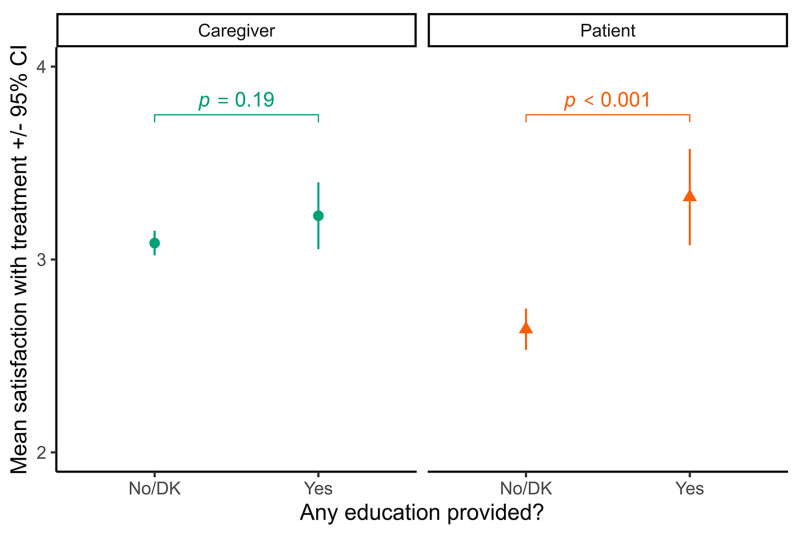
Correlation between caregiver/patient education and treatment satisfaction. There were no significant differences between caregivers reporting receiving disease-specific education (*n* = 48) compared to those who did not (*n* = 260). Adult patients who received any disease-specific education (*n* = 34) were significantly more satisfied with their treatments (estimated marginal mean adjusted for severity, EMM 3.32 (95% CI 3.09–3.56)) than those that did not receive any disease-specific education (*n* = 237, EMM 2.64, 95% CI 2.55–2.75, *p* < 0.001). DK means “don’t know” whether the provider discussed treatment priorities.

**Figure 5:**
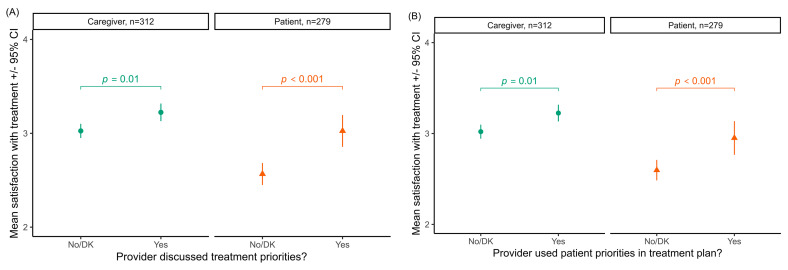
Treatment satisfaction when healthcare providers discussed treatment priorities with caregivers/patients (**A**) and when providers used those priorities to formulate the treatment plan (**B**). For both patients and caregivers, treatment satisfaction was significantly higher if healthcare providers discussed priorities and used those priorities to formulate the treatment plan. DK means “don’t know” whether the provider discussed treatment priorities.

Responses to questions related to patient engagement suggest there is little to no disease-specific education being offered to patients or their caregivers. Only 16% of caregivers reported being offered any training about AD and available treatments at their most recent AD-related medical care visit, while only 13% of adult patients reported being offered training. Adult patients who received any disease-specific education were more satisfied with their treatments (estimated marginal mean, EMM 3.32, 95% CI 3.09–3.56) than those that did not receive any disease-specific education (EMM 2.64, 95% CI 2.55–2.75, *p* < 0.001). There were no significant differences in the perception of disease control or treatment satisfaction in surveyed caregivers of pediatric patients with or without disease-specific education (Figure 4). Approximately 41% of caregivers reported being asked about their own or child’s priorities for care, while 33% of adult patients reported this question being asked during AD-related medical care appointments. For both patients and caregivers, treatment satisfaction was significantly higher if healthcare providers discussed priorities (Figure 5A) and used those priorities to formulate the treatment plan (Figure 5B). Respondents reported that specialists (dermatologists, pediatricians, and allergists) were significantly more likely than general practitioners to ask about respondents’ treatment priorities (severity-adjusted odds ratio = 1.61, 95% CI 1.04–2.52, *p* = 0.04).

## 4. Discussion

Here, we report the results of a 53-item survey covering demographics, healthcare provider information, financial burden, self-reported disease severity, burden of disease in terms of quality of life and willingness to exchange life years for improved health, and associations between disease-specific education and disease severity or treatment outcomes administered to an Argentinian population of adult AD patients and caregivers of pediatric AD patients. To our knowledge, no other studies have looked at health-related quality of life using the five-level EuroQol five-dimensional questionnaire (EQ-5D). To our knowledge, there have also not been surveys related to patient disease-specific education or shared patient/healthcare provider decision making and their effects on treatment satisfaction in Argentina. Anxiety/depression and pain/discomfort were the most impactful on respondents’ quality of life. Respondents reported financial burden including using savings or not purchasing food or other essentials to afford medical care. Findings indicate that greater emphasis should be placed during health care discussions on mental health burden, pain and discomfort, and financial burden in AD patients and their families. Other areas for improvement with regard to the patient experience would be to focus more on disease-specific education about disease management and treatment options, as well as shared decision making, taking patient priorities into consideration when creating treatment plans.

Patient disease-specific education and shared decision-making conversations may require further physician education first. A small study about physician perceptions of severity across countries with emerging economies included 50 dermatologists and pediatricians treating 147 children and adolescents with AD (ages 6 to 17) and reported that physicians tended to underestimate the disease severity when compared to validated SCORAD criteria [9]. In Argentina, 84% of physicians underestimated the disease severity vs. the pre-defined criteria in children with severe AD, which was slightly higher than the global average of 78% of physicians underestimating the disease severity [11]. A total of 74% of Argentinian physicians treating adolescents with severe disease underestimated the disease severity, but 87% of physicians treating adolescents with moderate disease matched the pre-clinically defined criteria, while only 13% overestimated the disease severity [11]. Physicians prioritized the elimination of itching and skin symptoms as their primary treatment objectives. Treatment approaches mostly included emollients, followed by highly potent topical corticosteroids. Fewer than 10% of patients across the countries with emerging economies in this study used biologics. Physicians reported being moderately to very concerned about exposing their pediatric patients to corticosteroids or other immunosuppressants [9]. This study may highlight the need to further educate physicians in countries with developing economies on how to objectively assess disease severity. As newer treatments become available in Argentina, physician education about the safety and efficacy of these new modalities will also be needed.

Fortunately, plans are beginning to be implemented worldwide regarding teaching physicians in underserved areas the most up-to-date AD information [12]. The Extension for Community Healthcare Outcomes (ECHO) project was developed in the United States to advance the education of clinicians worldwide on chronic, prevalent, complex diseases, including AD. The ECHO-AD program has been implemented in Argentina since 2019 and consists of the presentation of clinical cases by videoconference, virtual classes, and a permanently available open chat between healthcare professionals caring for patients with AD and a group of experts. The first study of the impact of this program in Argentina was published in 2022, revealing the significant improvement of physicians in the management of AD patients with an increased ability to interpret severity scores, employ phototherapy as a treatment option, and prescribe both classically utilized and emerging treatment options [12]. Though the majority of the 217 physicians who had participated in the program and survey were from Buenos Aires Province and Capital Federal, participants from all 20 provinces did respond to the survey about ECHO-AD. Physicians overwhelmingly (98%) said they would recommend the use of the ECHO-AD program to others, which is encouraging given the success of the program in improving physician understanding of the disease and, ultimately, improving patient outcomes [12]. With greater physician education, patients may also have access to more disease-specific education and improved shared patient/physician decision making about treatment options. We did not collect data about whether any of our survey respondents had physicians who had access to the ECHO-AD program. Future studies determining whether physician education about AD disease management leads to increased patient education about the disease and better shared decision making seem important.

Formal recommendations have been made to close AD knowledge gaps in Latin America including prioritizing funding for and urging for an increase in AD research in each region, creating a disease registry to systematize data collection, encouraging telehealth and web-based surveys, conducting health economic evaluations, unifying the diagnostic criteria being used across the nation, addressing the problem of the lack of access to specialists, particularly in rural parts of the countries, providing education on diagnosis and treatment that spans patients and caregivers to healthcare providers at all levels of care, creating a public awareness campaign, and supporting AD patient associations to advocate and raise awareness of AD [7]. These strategies are complex and will require financial support, but the ability to reduce the burden of disease across Latin America and drastically improve patient outcomes is high if even a subset of these approaches is implemented.

This study’s primary limitation is the requirement to have access to the Internet to complete the survey. AD patients and caregivers who found the survey in Argentina were likely already active in online AD communities, which may indicate that they are more connected to available AD knowledge compared to the general AD patient population in Argentina. Only 673 participants completed the survey, a relatively small sample size. The vast majority of survey respondents (caregivers and adult AD patients) were female. We suspect but do not know that caregivers of pediatric AD patients may be more commonly female. There was no difference between sex in the pediatric patient population on which the majority-female caregivers reported. A past report indicated that there are more female than male AD patients in Argentina [3], but there may be other reasons influencing the number of female patients who answered our survey. The limitation is that our results do not reflect well the experience of the adult male AD patient population or children of male caregivers. We did not include any survey questions about the overall level of education of the survey respondents. Education in this case only means whether the respondents were given access to additional information about their disease outside of the healthcare office visit and the duration (hours) of the education. Therefore, we cannot say whether overall education played a role in treatment satisfaction or disease control. This study is observational in nature, and, therefore, we can only discuss, but not assess, causative relationships. Lastly, this study represents a single-point-in-time picture of the AD patient experience, which may evolve as health equity focus changes around the globe.

In conclusion, we have reported the results from a survey of AD patients and caregivers in Argentina, highlighting the burden of disease with regard to quality of life and financial burden. We highlighted the importance of access to specialists, patient and caregiver disease-specific education on disease management and treatment options, and shared decision making for patients and caregivers to feel that their needs are being taken into consideration in making treatment recommendations. Our study adds to the scant available data about patient and caregiver experiences of AD in Argentina, reducing existing knowledge gaps and, to our knowledge, is the first to address disease-specific education and shared decision making. Our data lead us to recommend that disease-specific education and shared decision making should be priorities when forming care plans with AD patients in Argentina. Mental health concerns, pain and discomfort, and financial burden are the most important concerns/burdens for adult patients living with AD and caregivers of children with AD in Argentina and should be kept as priorities when forming care plans.

## 5. Key Points

Little is known about patients’ and caregivers’ experiences with AD in Argentina. A survey of 673 Argentinian AD patients and caregivers found that mental health, pain/discomfort, and financial worries are the highest burdens. The findings of the survey indicate that disease-specific education and shared patient/physician decision making should be priorities when forming care plans with Argentinian AD patients.

## Figures and Tables

**Table 1:** Demographics of individuals participating in the survey.

	**Caregiver (*n* = 339)**	**Adult Patient (*n* = 334)**	**Total (*n* = 673)**
Respondent age			
-No answer	64	2	66
-Mean (SD)	40.1 (7.6)	41.9 (13.2)	41.1 (11.0)
-Range			18–80
Respondent sex			
-Male	32 (9.4%)	29 (8.7%)	61 (9.1%)
-Female	306 (90.3%)	305 (91.3%)	611 (90.8%)
-Other	1 (0.3%)	0 (0.0%)	1 (0.1%)
	**Child Patient as Reported by Caregiver (*n* = 339)**	**Adult Patient (*n* = 334)**	**Total (*n* = 673)**
Subject age			
-No answer	66	2	68
-Mean (SD)	9.1 (5.0)	41.9 (13.2)	27.1 (19.4)
-Range	1–22	18–80	1–80
Subject sex			
-No answer	4	0	4
-Male	152 (45.4%)	29 (8.7%)	181 (27.1%)
-Female	182 (54.3%)	305 (91.3%)	487 (72.8%)
-Other	1 (0.3%)	0 (0.0%)	1 (0.1%)
Provider			
-No answer	3	5	8
-Dermatologist	215 (64.0%)	229 (69.6%)	444 (66.8%)
-Pediatrician	88 (26.2%)	2 (0.6%)	90 (13.5%)
-PCP/GP	3 (0.9%)	45 (13.7%)	48 (7.2%)
-Allergist	22 (6.5%)	29 (8.8%)	51 (7.7%)
-Derm. Nurse	0 (0.0%)	0 (0.0%)	0 (0.0%)
-NP	0 (0.0%)	0 (0.0%)	0 (0.0%)
-Other	8 (2.4%)	24 (7.3%)	32 (4.8%)
Disease severity			
-No answer	1	2	3
-Without symptoms	12 (3.6%)	7 (2.1%)	19 (2.8%)
-Mild	97 (28.7%)	126 (38.0%)	223 (33.3%)
-Moderate	181 (53.6%)	170 (51.2%)	351 (52.4%)
-Severe	48 (14.2%)	29 (8.7%)	77 (11.5%)
Insurance			
-Private insurance	151 (44.5%)	120 (35.9%)	271 (40.3%)
-Self insurance	175 (51.6%)	149 (44.6%)	324 (48.1%)
-Public insurance	38 (11.2%)	62 (18.6%)	100 (14.9%)

No answer means the number of survey respondents who did not answer the specific question. Caregiver alone means demographics of the adult caregiver only (also called respondent). Child Patient as Reported by Caregiver means that the answers were given by the caregiver but pertain to the child (also called subject). Adult Patient means that the adult patient answered the questions on their own behalf. PCP/GP is primary care physician/general practitioner; NP is nurse practitioner.

## Data Availability

Further information and raw data are available through contacting the corresponding author.
